# 3,4-Secocycloartane Triterpenoids from the Cones of *Pseudolarix amabilis*

**DOI:** 10.1007/s13659-020-00285-7

**Published:** 2021-01-03

**Authors:** Si-Jia Xiao, Bo Li, Zheng-Rui Huang, Wen-Lin Yuan, Ji Ye, Hui-Liang Li, Xi-Ke Xu, Yun-Heng Shen, Wei-Dong Zhang

**Affiliations:** 1 Department of Phytochemistry, School of Pharmacy, Naval Medical University (Second Military Medical University), Shanghai, 200433 China; 2 State Key Laboratory of New Drug and Pharmaceutical Process, Shanghai Institute of Pharmaceutical Industry, China State Institute of Pharmaceutical Industry, Shanghai, 201203 China; 3 Department of Applied Chemistry, Xi’an University of Technology, Xi’an, 710048 China

**Keywords:** *Pseudol arixamabilis*, Triterpenoids, 3,4-Secocycloartane, Pseudolactones A–D

## Abstract

**Abstract:**

Four new 3,4-secocycloartane triterpenoids, pseudolactones A–D (**1**–**4**), were isolated from the ethanol extract of the cones of *Pseudol arixamabilis*. Their structures were established by extensive 1D- and 2D-NMR experiments. The cones of *P. arixamabilis* are enriched in the ring-expanded or cleaved cycloartane triterpenoids. This work provides new insight into cycloartane triterpenoids from the cones of *P. arixamabilis*.

**Graphic Abstract:**

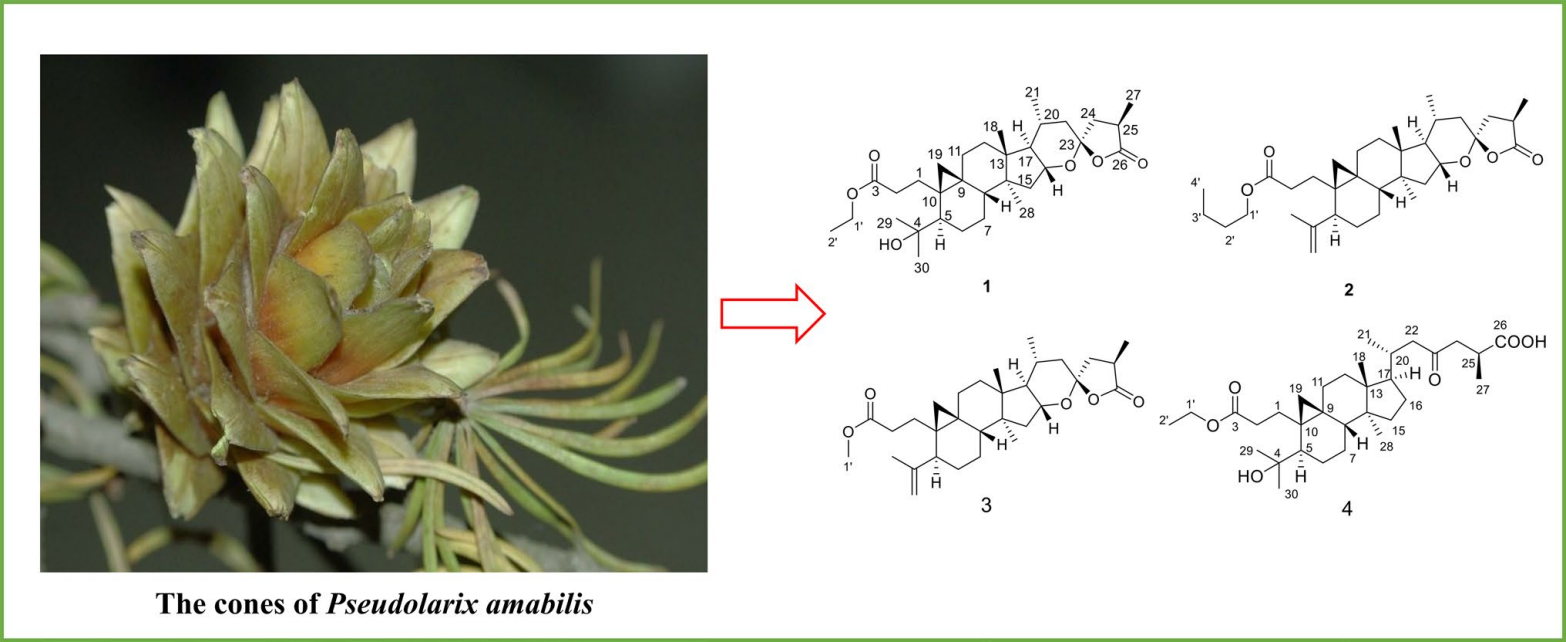

**Electronic supplementary material:**

The online version of this article (10.1007/s13659-020-00285-7) contains supplementary material, which is available to authorized users.

## Introduction

*Pseudolarix amabilis* is a plant indigenous to the south-east of China [1]. The root and trunk barks of it, known as ‘Tu Jin Pi’ in traditional Chinese medicine, have been used to treat skin diseases caused by fungal infection [2, 3]. Previous phytochemical studies on the root barks and seeds of *P. amabilis* revealed a variety of bioactive compounds with novel structures, with the main chemical constituents being pseudolaric acid analogous and triterpenoids [4–9]. Some of them, such as psedolaric acids A and B [10], have shown potent antimicrobial and cytotoxic activities. Peudolarolide B, a triterpene lactone, has shown potent cytotoxic activity [8]. Novel nortriterpenoid lactone, pseudolarenone [11], as well as triterpenoid–diterpenoid dimers [12], have also been reported from the cones of this plant. In this paper, we describe the isolation and structure elucidation of four new 3,4-secocycloartane triterpenoids from the cones of *P. amabilis*.

## Results and Discussion

**Fig. 1:**
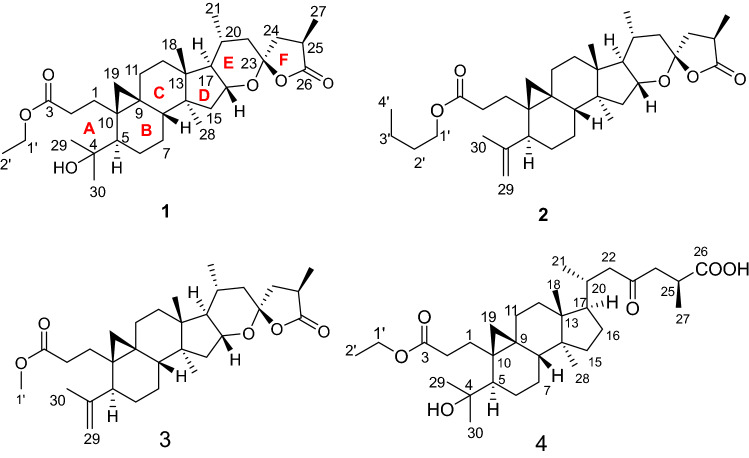
Chemical structures of **1**–**4**

The dried cones of *P. amabilis*, collected in Jiujiang, Jiangxi province, P. R. China, were extracted with 80% EtOH for three times at room temperature. The extract was separated by chromatography techniques to yield four new triterpenes, pseudolactones A–D (**1**–**4**) (Fig. 1). The structures of four new triterpenoids were determined by analysis of HRESIMS and NMR spectroscopic data.

Compound **1** corresponds to the molecular formula C_32_H_50_O_6_ as established by the hydrogen adduct ion peak at *m/z* 531.3678 [M + H]^+^ (calcd. 531.3680 for C_32_H_51_O_6_^+^) in HR-ESI–MS spectrum, indicative of 8 degrees of unsaturation.

The ^1^H NMR spectrum (Table 1) of **1** showed four tertiary (*δ*_H_ 1.00 (s), 1.17 (s), 1.18 (s), 1.08 (s)) and two secondary (*δ*_H_ 0.86 (d, *J* = 6.4 Hz), 1.19 (overlap)) methyls. Moreover, an oxygenated proton signal was observed at *δ*_H_ 4.05. In ^13^C NMR spectrum (Table 2), there existed 32 carbon resonances, which were sorted into seven methyls, eleven methylenes, six methines (including one oxygenated methane at *δ*_C_ 77.4), and eight quaternary carbons (including two ester carbonyls at *δ*_C_ 174.6 and 179.7, an oxygenated quaternary carbon at *δ*_C_ 76.1, and a ketal carbon at *δ*_C_ 107.3), by DEPT NMR spectrum. The above evidences, combined with the characteristic methylene protons at *δ*_H_ 0.51 (d, *J* = 4.4 Hz) and 0.70 (d, *J* = 4.4 Hz) as well as the carbon resonances at *δ*_C_ 32.4 (t), 21.8 (s), and 27.6 (s), revealed a cyclopropyl motif. The diagnostic chemical shifts for two ester carbonyls at *δ*_C_ 174.6 (C-3) and 179.7 (C-26), one oxygenated methine at *δ*_C_ 77.4, and the ketal carbon at *δ*_C_ 107.3, implied that compound **1** might be a 3,4-secocycloartane triterpenoid with a unique 16,23-epoxy-23,26-spirolactone side chain.

**Table 1:** ^1^H NMR (400 MHz) spectroscopic data for compounds **1**–**4** in CDCl_3_ (*J* in Hz)

No.	*δ*_H_ (*δ* in ppm, *J* in Hz)			
	**1**	**2**	**3**	**4**
1	2.17 m	1.35 overlap,	1.34 m	2.29 ddd (4.0, 9.2, 15.6)
	2.59 ddd (6.0, 10.0, 16.0)	2.03 dd (2.8, 6.0, 16..0)	2.02 dd (6.0, 16.0)	2.60 ddd (5.6, 10.0, 15.6)
2	1.31 overlap	2.22 m	2.21 m	1.36 m
	2.64 ddd (6.0, 10.8, 16.4)	2.49 ddd (6.0, 12.0, 15.6)	2.48 ddd (6.0, 12.0, 15.6)	2.15 m
3				
4				
5	1.27 m	2.41 overlap	2.38 overlap	1.85 m
6	0.63 m	1.08 m	1.05 overlap	0.69 m
	1.69 m	1.51 m	1.48 m	1.73 m
7	0.96 m	1.10 m	1.05 overlap	1.23 m
	1.23 m	1.26 overlap	1.23 m	1.28 m
8	1.82 m	1.53 m	1.50 m	1.30 m
9				
10				
11	2.10 m	2.17 m	2.13 m	1.20 m
	1.20 overlap	1.26 overlap	1.26 m	1.87 m
12	1.40 m	1.68 m	1.57 m	1.64 overlap
	1.14 m	1.58 overlap	1.66 m	1.40 overlap
13				
14				
15	1.21 overlap	1.23 overlap	1.20 m	1.33 m
	1.72 m	1.79 dd (11.2, 14.0)	1.75 dd (11.2, 12.4)	1.62 overlap
16	4.05 overlap	4.09 td (5.6, 11.2)	4.06 m	1.39 overlap
				2.64 ddd (4.0, 8.8, 15.6)
17	1.43 m	1.47 m	1.45 m	1.63 overlap
18	1.00 s	1.04 s	1.01 s	1.00 s
19	0.51 d (4.4)	0.42 d (4.8)	0.39 d (4.8)	0.57 d (4.4)
	0.70 d (4.4)	0.78 d (4.8)	0.76 d (4.8)	0.68 d (4.4)
20	2.03 m	2.10 m	2.05 m	2.02 m
21	0.86 d (6.4)	0.89 d (6.8)	0.87 d (6.4)	0.86 d (6.0)
22	1.35 m	1.40 m	1.38 dd (2.4, 14.0)	2.18 m
	1.86 m	1.90 dd (4.0, 14.0)	1.87 dd (4.0, 14.0)	2.51 m
23				
24	1.66 m	1.70 m	1.69 d (1.2)	2.47 dd (4.8, 17.2)
	2.38 dd (8.4, 12.8)	2.40 overlap	2.38 overlap	2.93 dd (12.4, 17.2)
25	2.87 m	2.93 m	2.91 m	2.96 m
26				
27	1.19 overlap	1.25 d (7.2)	1.22 d (7.2)	1.22 d (9.6)
28	1.17 s	1.12 s	1.09 s	0.92 s
29	1.18 s	4.73 dd (1.6, 2.0)	4.71 dd (2.0, 1.6)	1.25 s
		4.80 d (2.0)	4.77 d (2.0)	
30	1.08 s	1.67 s	1.65 s	1.21 s
1′	4.05 q (6.8)	4.03 t (6.8)	3.61 s	4.11 q (6.8)
2′	1.19 overlap	1.59 overlap		1.25 t (6.8)
3′		1.37 overlap		
4′		0.93 t (7.6)

**Table 2:** ^13^C (100 MHz) NMR spectroscopic data of compounds **1**–**4** in CDCl_3_

No.	*δ*_C_ (*δ* in ppm)			
	**1**	**2**	**3**	**4**
1	32.2 (t)	28.6 (t)	28.6 (t)	32.3 (t)
2	29.5 (t)	31.6 (t)	31.4 (t)	26.4 (t)
3	174.6 (s)	174.0 (s)	174.3 (s)	174.8 (s)
4	76.1 (s)	149.2 (s)	149.2 (s)	76.3 (s)
5	48.1 (d)	46.1 (d)	46.1 (d)	45.2 (d)
6	25.0 (t)	27.5 (t)	27.5 (t)	25.4 (t)
7	26.0 (t)	25.5 (t)	25.5 (t)	25.7 (t)
8	45.4 (d)	47.9 (d)	47.8 (d)	48.6 (d)
9	21.8 (s)	20.8 (s)	20.8 (s)	22.6 (s)
10	27.6 (s)	27.7 (s)	27.6 (s)	26.5 (s)
11	26.7 (t)	26.9 (t)	26.9 (t)	28.3 (t)
12	31.1 (t)	31.0 (t)	31.0 (t)	33.0 (t)
13	43.5 (s)	43.8 (s)	43.8 (s)	44.9 (s)
14	47.3 (s)	47.4 (s)	47.4 (s)	49.0 (s)
15	41.1 (t)	40.8 (t)	40.8 (t)	35.9 (t)
16	77.4 (d)	77.3 (d)	77.2 (d)	30.2 (t)
17	54.8 (d)	54.8 (d)	54.8 (d)	52.3 (d)
18	19.4 (q)	19.3 (q)	19.3 (q)	18.5 (q)
19	32.4 (t)	31.6 (t)	31.6 (t)	30.9 (t)
20	29.9 (d)	30.0 (d)	30.0 (d)	33.0 (d)
21	19.1 (q)	19.2 (q)	19.2 (q)	19.2 (q)
22	44.1 (t)	44.2 (t)	44.1 (t)	50.2 (t)
23	107.3 (s)	107.3 (s)	107.3 (s)	209.3 (s)
24	42.6 (t)	42.7 (t)	42.7 (t)	46.4 (t)
25	34.1 (d)	34.2 (d)	34.1 (d)	34.4 (d)
26	179.7 (s)	179.8 (s)	179.7 (s)	180.8 (s)
27	14.9 (q)	14.9 (q)	14.9 (q)	16.9 (q)
28	31.7 (q)	23.1 (q)	23.0 (q)	19.5 (q)
29	25.8 (q)	111.6 (t)	111.6 (t)	31.6 (q)
30	23.1 (q)	19.7 (q)	19.7 (q)	26.2 (q)
1′	60.2 (t)	64.2 (t)	51.5 (q)	60.3 (t)
2′	14.1 (q)	30.6 (t)		14.2 (q)
3′		19.1 (t)		
4′		13.7 (q)		

**Fig. 2:**
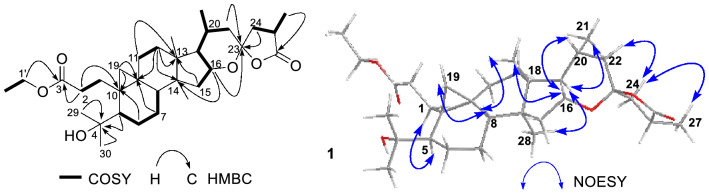
Key ^1^H-^1^H COSY, HMBC, and NOESY correlations of compound **1**

Comparison of the NMR spectroscopic data of compound **1** with those of known pseudolarolide C [8] indicated that they were structurally quite similar except that an additional ethoxy in compound **1** (*δ*_H_ 1.19 (3H), 4.05 (2H); *δ*_C_ 14.1, 60.2) replaced the methoxyl in pseudolarolide C, which was confirmed by key ^1^H-^1^H COSY correlations (Fig. 2) of H_2_-1′/H_3_-2′ and HMBC correlation from H_2_-1′ (*δ*_H_ 4.05) to carboxylic carbon C-3 (*δ*_C_ 174.6 ppm). The structure of **1** was further evidenced by key ^1^H-^1^H COSY correlations of H_2_-2/H_2_-1, H-5/H_2_-6/H_2_-7/H-8, H_2_-11/H_2_-12, H_2_-15/H-16/H-17/H-20 (H_3_-21)/H_2_-22 together with key HMBC correlations (Fig. 2) from H_2_-2, H_2_-19, H_2_-6 to C-10 (*δ*_C_ 27.6), from H_2_-11, H-15 to C-13 (*δ*_C_ 43.5), from H_2_-12, H-16 to C-14 (*δ*_C_ 47.3), from H_2_-22, H-16, H_2_-24 to C-23 (*δ*_C_ 107.3), and H_3_-27 to C-26 (*δ*_C_ 179.7). The relative configurations of **1** were elucidated to be identical with those of pseudolarolide C on the basis of the REOSY correlations (Fig. 2) of H-19 with H-8, H-8 with CH_3_-18, CH_3_-18 with H-16, H-16 with H-20, H-17 with CH_3_-28 and CH_3_-21, H-22 with H-24, and H-24 with CH_3_-27. In cycloartane triterpenoids, the C-20 position of the 17-side chain is usually *R*-configuration. From the biogenetic point of view, the C-20 position of **1** should be *R*-configuration, too. Zhao et al. reported a serial of similar 16,23-epoxy-26(23)-olide-3,4-secocycloartan-3-oic acid esters, and determined their absolute configurations by single crystal X-ray diffraction [13]. By comparing the Cotton effect (− 0.7 at 223 nm) of **1** in ECD spectrum with those known compounds [13], in combination with analysis of chemical shifts, the absolute configurations of C-23 and C-25 positions of **1** were proposed to be *S*- and *R*-configuration, respectively. Thus, the structure of **1** was elucidated as (20*R*,23*S*,25*R*)-4-hydroxy-16,23-epoxy-26(23)-olide-3,4-secocycloartan-3-oic acid ethyl ester, and named pseudolactone A.

Compound **2** was obtained as colorless oil. Its molecular formula was determined as C_34_H_54_O_6_, by HR-ESI–MS spectrum. The ^1^H and ^13^C NMR spectroscopic data (Tables 1 and 2) of **2** showed typical signals similar to those of **1**, including two ester carbonyls (*δ*_C_ 174.0, 179.8), a ketal carbon at *δ*_C_ 107.3 (C-23), an oxygenated methine at *δ*_C_ 77.3 (C-16), a cyclopropyl at *δ*_H_ 0.42 (d, *J* = 4.8 Hz), 0.78 (d, *J* = 4.8 Hz) and *δ*_C_ 31.6 (t), one oxygen-bearing proton at *δ*_H_ 4.09 (td, *J* = 5.6, 11.2 Hz) and *δ*_C_ 77.3 (d), one oxygenated methylene at *δ*_H_ 4.03 (t, *J* = 6.8 Hz) and *δ*_C_ 64.2 (t), three singlet methyls at *δ*_H_ 1.04 (s), 1.12 (s), and 1.67 (s), two doublet methyls at *δ*_H_ 0.89 (d, *J* = 6.8 Hz) and 1.25 (d, *J* = 7.2 Hz), one triplet methyl at *δ*_H_ 0.93 (t, *J* = 7.6 Hz). These information revealed that compound **2** possessed the similar 16,23-epoxy-26(23)-olide-3,4-secocycloartane skeleton to that of **1**. The main differences between compounds **2** and **1** are that the resonance signals for one terminal double bond (*δ*_H_ 4.73 (dd, *J* = 1.6, 2.0 Hz), 4.80 (d, *J* = 2.0 Hz); *δ*_C_ 149.2 (s), 111.6 (t)), and for one butoxy (*δ*_H_ 4.03, 1.59, 1.37, 0.93; *δ*_C_ 64.2 (t), 30.6 (t), 19.1 (t), 13.7 (q)) in **2**, took place of the signals for the oxygenated quaternary carbon at *δ*_C_ 76.1 (s, C-4) and the methyl at *δ*_H_ 1.17 (s) and *δ*_C_ 25.8 (q), as well as for the ethoxy at *δ*_H_ 4.05, 1.19 and *δ*_C_ 60.2 (t), 14.1 (q).

**Fig. 3:**
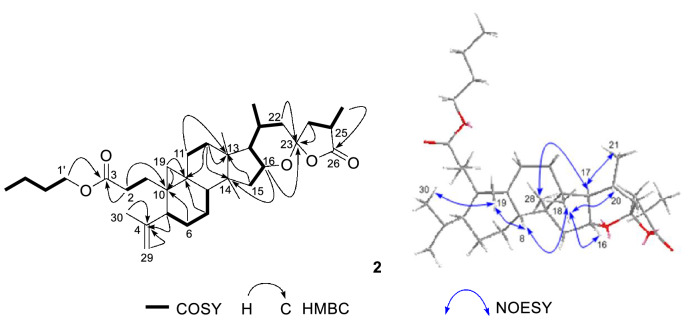
Key ^1^H-^1^H COSY, HMBC, and NOESY correlations of compound **2**

In ^1^H-^1^H COSY spectrum (Fig. 3), a butoxy was determined on the basis of the correlations of H_2_-1′/H_2_-2′/H_2_-3′/H_3_-4′. The butoxy was linked to C-3 through key HMBC cross peak (Fig. 3) from H_2_-1′ (*δ*_H_ 4.03) to the ester carbonyl at *δ*_C_ 174.0. The terminal double bond was evidenced to be placed between C-4 and C-29 as shown by key HMBC correlations (Fig. 3) from H-5 (*δ*_H_ 2.41), H_2_-29 (*δ*_H_ 4.73 and 4.80), CH_3_-30 (*δ*_H_ 1.67) to C-4 (*δ*_C_ 149.2). In REOSY spectrum of **2**, the observation of key NOE correlations (Fig. 3) of H-19 with CH_3_-30, H-8 (*δ*_H_ 1.53), of CH_3_-18 (*δ*_H_ 1.04) with H-8, H-16 (*δ*_H_ 4.09) and H-20 (*δ*_H_ 2.10), of H-17 (*δ*_H_ 1.47) with CH_3_-28 (1.12) and CH_3_-21 (*δ*_H_ 0.89), suggested that the relative configuration of **2** was identical to that of compound **1**. From structure, compound **2** could be considered as a dehydrated analogue of **1**, implying that two compounds may share same configuration. Also, compound **2** had a negative Cotton effect (− 1.5) at 201 nm. Consequently, the structure of **2** was identified as (20*R*,23*S*,25*R*)- 16,23-epoxy-26(23)-olide-3,4-secocycloartan-4(29)-en-3-oic acid *n*-butyl ester, and named pseudolactone B.

The molecular formula of compound **3** was assigned as C_31_H_46_O_5_ with nine degrees of unsaturation, based on the hydrogen adduct ion [M + H]^+^ at *m*/*z* 499.3414 (calcd. 499.3418 for C_31_H_47_O_5_^+^). Its molecular weight is 42 Da less than that of **2**. The ^1^H and ^13^C NMR spectra (Tables 1 and 2) were quite close to those of **2**. The only difference was that a methoxyl at *δ*_H_ 3.61 and *δ*_C_ 51.5 was observed in **3**, instead of those signals for the butoxy (*δ*_H_ 4.03, 1.59, 1.39, 0.93; *δ*_C_ 64.2 (t), 30.6 (t), 19.1 (t), 13.7 (q)) in **2**. Key HMBC correlation from the methoxyl proton at *δ*_H_ 3.61 to the ester carbonyl at *δ*_C_ 174.3 further confirmed the above deduction, and established the structure of **3** as (20*R*,23*S*,5*R*)-16,23-epoxy-26(23)-olide-3,4-secocycloartan-4(29)-en-3-oic acid methyl ester, and given the name pseudolactone C.

Compound **4** had a molecular formula C_32_H_52_O_6_ with seven degrees of unsaturation, as evidenced by positive HR-ESI–MS at *m*/*z* 555.3674 [M + Na]^+^. The ^1^HNMR spectroscopic data (Table 1) of **4** exhibited four singlet methyls at *δ*_H_ 1.00 (s), 0.92 (s), 1.25 (s), and 1.21 (s), two doublet methyls at *δ*_H_ 0.86 (d, *J* = 6.0 Hz) and 1.22 (d, *J* = 9.6 Hz), one triplet methyl at *δ*_H_ 1.25 (t, *J* = 6.8 Hz), and an oxygenated methylene at *δ*_H_ 4.11 (2H, q, *J* = 6.8 Hz). In addition, a typical AB coupling system was observed at *δ*_H_ 0.57 (1H, d, *J* = 4.4 Hz) and 0.68 (1H, d, *J* = 4.4 Hz). In ^13^C NMR spectrum (Table 2), thirty-two carbon resonances were observed, including 7 methyls (*δ*_C_ 18.5, 19.2, 16.9, 19.5, 31.6, 26.2, 14.2), 12 methylenes (including one oxygenated methylene at *δ*_C_ 60.3), 5 methines, and 8 quaternary carbons (including one ester carbonyl at *δ*_C_ 174.8, one carboxyl at *δ*_C_ 180.8, one ketone carbonyl at *δ*_C_ 209.3, and one oxygenated quaternary carbon at *δ*_C_ 76.3). Deducting three degrees of unsaturation accounted for one ketone carbonyl and two carboxylic carbon, the remaining four degrees of unsaturation were indicative of the tetracyclic ring system of **4**. The NMR spectroscopic data (Tables 1 and 2) of **4** quite resemble those of known compound pseudolarnoid G ((25S)-4-hydroxy-3,4-seco-cycloartan-23-one-3,26-dioic acid methyl), previously reported from the seeds of the tilted plant [13]. The main differences are that compound **4** had an ethoxy ester and one carboxyl functionalities, while pseudolarnoid G had two methyl ester functionalities.

**Fig. 4:**
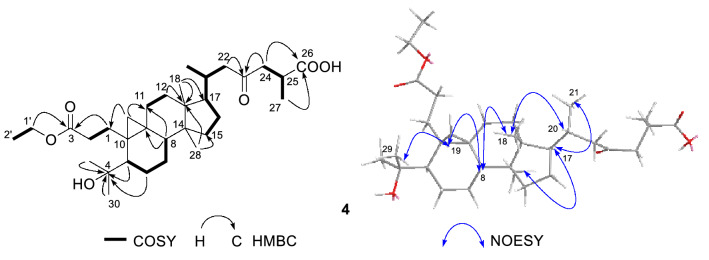
Key ^1^H-^1^H COSY, HMBC, and NOESY correlations of compound **4**

The diagnostic chemical shifts at *δ*_C_ 174.8 (ester carbonyl) and *δ*_C_ 76.3 (s) were indicative of oxidative cleavage of ring A between C-3 and C-4, and formed an ethyl ester at C-3 and an oxygenated isopropyl moiety at C-4. The deduction was evidenced by key ^1^H-^1^H COSY correlation (Fig. 4) between H_2_-1′ (*δ*_H_ 4.11) and H_2_-2′ (*δ*_H_ 1.25), and between H_2_-1 and H_2_-2, together with HMBC correlations (Fig. 4) of H_2_-1 and H_2_-1′ with C-3 (*δ*_C_ 174.8), of CH_3_-29 (*δ*_H_ 1.25), CH_3_-30 (*δ*_H_ 1.21), H-5 (*δ*_H_ 1.85), H_2_-6 (*δ*_H_ 0.69, 1.73) with the oxygenated quaternary carbon at *δ*_C_ 76.3 (C-4). Also, the ketone carbonyl at *δ*_C_ 209.3 was assigned to be C-23 on the basis of HMBC correlations from H_2_-22 and H_2_-24 to the ketone carbonyl. Key HMBC cross-peaks of H_2_-24 and CH_3_-27 (*δ*_H_ 1.22 (d, *J* = 9.6 Hz) supported the attribution of C-26 carboxyl. In the ROESY spectrum, key NOE correlations (Fig. 4) of H-20 with CH_3_-18, of H-8 (*δ*_H_ 1.30) with CH_3_-18 and H-19, and of CH_3_-29 with H-19 indicated that H-8, CH_3_-18, CH_2_-19, H-20, and 4-hydroxyl isopropyl are *β*-oriented, whereas H-17 and CH_3_-28 were *α*-oriented based on the NOE cross-peak of H-17 with CH_3_-28. With regard to the absolute configurations of two chiral centers (C-20 and C-25) at C-17 side chain, Zhao et al. had ever determined the absolute configurations of C-20 and C-25 of similar compound pseudolarnoid G as *R* and *S*, respectively, by single crystal X-ray diffraction. The ECD spectrum of compound **4** showed a negative Cotton effect − 1.5 at 285 nm, in accordance with that of pseudolarnoid G (− 1.23 (283 nm)), revealed that compound **4** had same absolute configuration as that of pseudolarnoid G. Therefore, the structure of **4** was identified as (20*R*,25*S*)-4-hydroxy-23-oxo-3,4-secocycloartan-26-oic acid-3-ethyl ester, and named pseudolactone D.

## Experimental Section

### General Experimental Procedures

Optical rotations were measured with a Perkin–Elmer 341 polarimeter. IR spectra were recorded with a Bruker Vector-22 Spectrophotometer with KBr discs. NMR spectra were recorded with Bruker *DRX-400* spectrometer (400 MHz). The chemical shifts (*δ*) are given in ppm with TMS as internal standard and coupling constants (*J*) are given in Hz. MS spectra were recorded with a *Agilent MSD-Trap-XCT* (for ESI) and Waters Micro-mass Q-TOF mass spectrometer (for HR-ESI–MS), in *m/z*. Column chromatographic separations were carried out by using silica gel (200–300 mesh; *Marine Chemical Factory*, Qingdao, P. R. China), Sephadex LH-20 (*Pharmacia Fine Chemicals*, Piscataway, NJ, USA) as packing material. TLC was carried out on precoated silica gel GF 254 plates (*Yantai Chemical Industrials*) and the TLC spots were viewed at 254 nm and visualized by using 10% sulfuric acid in alcohol containing 10 mg/mL vanillin.

### Plant Material

The cones of *P. amabilis* were collected in Jiu Jiang, Jiangxi province, P. R. China, in October 2010, and authenticated by Prof. Han-Ming Zhang of Second Military Medical University. A voucher specimen (No. 20101015) is deposited in School of Pharmacy, Second Military Medical University.

### Extraction and Isolation

The air-dried cones (12.0 kg) of *P. amabilis* were ground into powder and extracted with 80% EtOH for four times at room temperature to give a crude extract, which was further partitioned with petroleum ether (60–90 °C) (PE), CHCl_3_, and EtOAc, successively. The CHCl_3_-soluable extract was subjected to a silica gel column chromatography (CC) eluting with a gradient PE/EtOAc (from 30:0 to 0:1) to obtain eight fractions 1–8. Fraction 2 (58 g) was chromatographed over RP-18 column eluting with MeOH/H_2_O (from 3:7 to 10:0) to afford five subfractions. Subfraction 2 was further chromatographed on a silica gel column (CH_2_Cl_2_/PE, from 0:20 to 1:0, and MeOH) and purified by preparative TLC (cyclohexane/CH_2_Cl_2_/EtOAc, 20:1:1) to afford **2** (20 mg) and **3** (20 mg). Subfraction 4 was separated by repeated column chromatography on Sephadex LH-20 (CHCl_3_/MeOH, 1:1, and MeOH), and then purified by preparative TLC (cyclohexane/CH_2_Cl_2_/EtOAc, 15:1:1) to yield **1** (10 mg). Fraction 7 (70 g) was separated by RP-18 CC (MeCN/H_2_O, from 2:8 to 10:0) to afford 6 subfractions. Subfraction 3 was further chromatographed on a silica gel column (CHCl_3_/MeOH from 50:1 to 0:1) and purified by preparative TLC (PE/EtOAc/MeOH, 20:1:0.1) to afford **4** (8 mg).

#### Pseudolactone A (**1**)

Colorless oil, [*α*] 45.1 (*c* = 0.37, CH_2_Cl_2_). CD (*c* = 2.83 mmol/L, CH_3_CN, 20 °C) nm (Δε) 223 (-0.7). IR (KBr) *ν*_max_ 1731, 1778, 2964 cm^−1^. For ^1^H and ^13^C NMR data (400 MHz, CDCl_3_), see Tables 1 and 2. ESI–MS: *m*/*z* 553.5 [M + Na]^+^, 529.3 [M − H]^−^. HR-ESI–MS: *m*/*z* 531.3678 [M + H]^+^ (calcd C_32_H_51_O_6_^+^, 531.3680).

#### Pseudolactone B (**2**)

Colorless oil, [*α*] 51.6 (*c* = 0.24, CH_2_Cl_2_). CD (*c* = 2.11 mmol/L, CH_3_CN, 20 °C) nm (Δε) 201 (− 1.5). IR (KBr) *ν*_max_ 1727, 1770, 2974 cm^−1^. For ^1^H (400 MHz, CDCl_3_) and ^13^C NMR data (100 MHz, CDCl_3_), see Tables 1 and 2. ESI–MS: *m*/*z* 563.2 [M + Na]^+^, 539.1 [M − H]^−^. HRESIMS: *m*/*z* 541.3902 [M + H]^+^ (calcd C_34_H_53_O_5_^+^, 541.3888).

#### Pseudolactone C (**3**)

Colorless oil, [*α*] 51.1 (*c* = 0.23, CH_2_Cl_2_). CD (*c* = 2.61 mmol/L, CH_3_CN, 20 °C) nm (Δε) 201 (− 1.6). IR (KBr) *ν*_max_ 1737, 1778, 2964 cm^−1^. For ^1^H (400 MHz, CDCl_3_) and ^13^C NMR data (100 MHz, CDCl_3_), sees Tables 1 and 2. ESIMS: *m*/*z* 521.2 [M + Na]^+^. HR-ESI–MS: *m*/*z* 499.3414 [M + H]^+^ (calcd C_31_H_47_O_5_^+^, 499.3418).

#### Pseudolactone D (**4**)

White amorphous powder, [*α*] 51.6 (*c* = 0.23, CH_2_Cl_2_). CD (*c* = 1.88 mmol/L, CH_3_CN, 20 °C) nm (Δε) 210 (− 1.9), 285 (− 1.5). IR (KBr) *ν*_max_ 1712, 1735, 2873, 2925, 2962, 3440 cm^−1^. For ^1^H (400 MHz, CDCl_3_) and ^13^C NMR data (100 MHz, CDCl_3_), see Tables 1 and 2. ESI–MS: *m*/*z* 531.4 [M − H]^−^. HR-ESI–MS *m*/*z* 555.3674 [M + Na]^+^ (calcd C_32_H_52_O_6_Na^+^, 555.3656).

## Electronic supplementary material

Below is the link to the electronic supplementary material.

Supplementary file1 (DOCX 1321 KB)
