# Supplementary material for: 3,4-Secocycloartane Triterpenoids from the Cones of Pseudolarix amabilis
Source: Nat Prod Bioprospect. 2021 Jan 3;11(1):119–26. doi: 10.1007/s13659-020-00285-7 (PMC7933301; doi:10.1007/s13659-020-00285-7)
Supplement: Supplementary file 1 — Supplementary file1 (DOCX 1321 KB) [file 13659_2020_285_MOESM1_ESM.docx]

**Supplementary Information**

**3,4-secocycloartane triterpenoids from the cones of *Pseudolarix amabilis***

**Si-Jia Xiao^1, ±^ ·Bo Li^1,2, ±^ ·Zheng-Rui Huang^3^ ·Wen-Lin Yuan^1^ ·Ji Ye^1^ ·Hui-Liang Li^1^ · Xi-Ke Xu^1^ ·Yun-Heng Shen^1,^* ·Wei-Dong Zhang ^1, 2,^[[1]](#footnote-1)^*^**

***This paper is dedicated to the memorial of Professor Jun Zhou***

^1^ Department of Phytochemistry, School of Pharmacy, Naval Medical University (Second Military Medical University), Shanghai 200433, China

^2^ State Key Laboratory of New Drug and Pharmaceutical Process, Shanghai Institute of Pharmaceutical Industry, China State Institute of Pharmaceutical Industry, Shanghai 201203, China

^3^ Department of Applied Chemistry, Xi’an University of Technology, Xi’an 710048, China

**Electronic supplementary material** The online version of this article (<https://doi.org/>) contains supplementary material, which is available to authorized users.

| **No.** | **Content** | **Page** |
| --- | --- | --- |
| 1 | Figure S1. ^1^H NMR spectrum of compound **1** | 1 |
| 2 | Figure S2. ^13^C NMR spectrum of compound **1** | 1 |
| 3 | Figure S3. DEPT spectrum of compound **1** | 2 |
| 4 | Figure S4. ^1^H-^1^H COSY spectrum of compound **1** | 2 |
| 5 | Figure S5. HSQC spectrum of compound **1** | 3 |
| 6 | Figure S6. HMBC spectrum of compound **1** | 3 |
| 7 | Figure S7. NOESY spectrum of compound **1** | 4 |
| 8 | Figure S8. ^1^H NMR spectrum of compound **2** | 4 |
| 9 | Figure S9. ^13^C NMR spectrum of compound **2** | 5 |
| 10 | Figure S10. DEPT spectrum of compound **2** | 5 |
| 11 | Figure S11. ^1^H-^1^H COSY spectrum of compound **2** | 6 |
| 12 | Figure S12. HSQC spectrum of compound **2** | 6 |
| 13 | Figure S13. HMBC spectrum of compound **2** | 7 |
| 14 | Figure S14. NOESY spectrum of compound **2** | 7 |
| 15 | Figure S15. ^1^H NMR spectrum of compound **3** | 8 |
| 16 | Figure S16. ^13^C NMR spectrum of compound **3** | 8 |
| 17 | Figure S17. DEPT spectrum of compound **3** | 9 |
| 18 | Figure S18. ^1^H-^1^H COSY spectrum of compound **3** | 9 |
| 19 | Figure S19. HSQC spectrum of compound **3** | 10 |
| 20 | Figure S20. HMBC spectrum of compound **3** | 10 |
| 21 | Figure S21. NOESY spectrum of compound **3** | 11 |
| 22 | Figure S22. ^1^H NMR spectrum of compound **4** | 11 |
| 23 | Figure S23. ^13^C NMR spectrum of compound **4** | 12 |
| 24 | Figure S24. DEPT spectrum of compound **4** | 12 |
| 25 | Figure S25. ^1^H-^1^H COSY spectrum of compound **4** | 13 |
| 26 | Figure S26. HSQC spectrum of compound **4** | 13 |
| 27 | Figure S27. HMBC spectrum of compound **4** | 14 |
| 28 | Figure S28. NOESY spectrum of compound **4** | 14 |

**Figure S1.** ^1^H NMR spectrum of compound **1**

**Figure S2.** ^13^C NMR spectrum of compound **1**

**Figure S3.** DEPT spectrum of compound **1**

**Figure S4.** ^1^H-^1^H COSY spectrum of compound **1**

**Figure S5.** HSQC spectrum of compound **1**

**Figure S6.** HMBC spectrum of compound **1**

**Figure S7.** NOESY spectrum of compound **1**

**Figure S8.** ^1^H NMR spectrum of compound **2**

**Figure S9.** ^13^C NMR spectrum of compound **2**

**Figure S10.** DEPT spectrum of compound **2**

**Figure S11.** ^1^H-^1^H COSY spectrum of compound **2**

**Figure S12.** HSQC spectrum of compound **2**

**Figure S13.** HMBC spectrum of compound **2**

**Figure S14.** NOESY spectrum of compound **2**

**Figure S15.** ^1^H NMR spectrum of compound **3**

**Figure S16.** ^13^C NMR spectrum of compound **3**

**Figure S17.** DEPT spectrum of compound **3**

**Figure S18.** ^1^H-^1^H COSY spectrum of compound **3**

**Figure S19.** HSQC spectrum of compound **3**

**Figure S20.** HMBC spectrum of compound **3**

**Figure S21.** NOESY spectrum of compound **3**

**Figure S22.** ^1^H NMR spectrum of compound **4**

**Figure S23.** ^13^C NMR spectrum of compound **4**

**Figure S24.** DEPT spectrum of compound **4**

**Figure S25.** ^1^H-^1^H COSY spectrum of compound **4**

**Figure S26.** HSQC spectrum of compound **4**

**Figure S27.** HMBC spectrum of compound **4**

**Figure S28.** NOESY spectrum of compound **4**

1. *** Yun-Heng Shen (**[**shenyunheng@hotmail.com**](mailto:shenyunheng@hotmail.com)**)**

   **Wei-Dong Zhang(**[**wdzhangy@hotmail.com**](mailto:wdzhangy@hotmail.com)**)**

   **^±^ These authors contributed equally to this work** [↑](#footnote-ref-1)
